# Prediction of Seat Belt Use Behavior among Adolescents Based on the Theory of Planned Behavior

**DOI:** 10.34172/jrhs.2021.71

**Published:** 2021-12-06

**Authors:** Fatemeh Malekpour, Babak Moeini, Leili Tapak, Homayoun Sadeghi-Bazargani, Forouzan Rezapur-Shahkolai

**Affiliations:** ^1^Department of Public Health, School of Public Health, Hamadan University of Medical Sciences, Hamadan, Iran; ^2^Social Determinants of Health Research Center, Hamadan University of Medical Sciences, Hamadan, Iran; ^3^Department of Biostatistics, School of Public Health, Hamadan University of Medical Science, Hamadan, Iran; ^4^Noncommunicable Diseases Research Center, Hamadan University of Medical Science, Hamadan, Iran; ^5^Road Traffic Injury Research Center, Tabriz University of Medical Science, Tabriz, Iran; ^6^Research Center for Health Sciences, Hamadan University of Medical Sciences, Hamadan, Iran

**Keywords:** Car Occupant, Health Promotion, Injury Prevention, Safe Behavior, Safety Promotion, School Student

## Abstract

**Background:** Road Traffic Injuries (RTIs) are the important causes of unintentional injuries and deaths. In this respect, seat belt wearing is an influential factor in reducing the mortality and severity of road traffic injuries. The rate of seat belt use among is lower adolescents, compared to adults. The present study aimed to investigate the influential factors on seat belt-wearing behavior among adolescent students as car occupants based on the Theory of Planned Behavior (TPB).

**Study design:** A cross-sectional design.

**Methods:** This study was conducted among 952 adolescent students studying in grades 7, 8, and 9 in the schools of Tabriz, Iran, in the 2019-20 academic year. A researcher-made questionnaire was designed based on TPB for data collection, the validity and reliability of which have been confirmed.

**Results:** The results indicated that the rate of seat belt use in the front seat inside the city was lower than that outside the city. Regarding TPB constructs, perceived behavioral control (β=0.137; 95% CI: 0.006, 0.013; *P*<0.001), subjective norm (β=0.313; 95% CI: 0.021, 0.032; *P*<0.001), and attitude (β=0.322; 95% CI: 0.034, 0.053; *P*<0.001) had a significant and positive relationship with the intention of seat belt-wearing behavior. Moreover, the behavioral intention (β=0.571; 95% CI: 0.62, 0.64; *P*<0.001) had a significantly positive relationship with seat belt-wearing behavior.

**Conclusion:** The Theory of Planned Behavior is appropriate to determine predictor factors of seat belt-wearing behavior among adolescent students as car occupants. In addition, the results of the present study may provide a theoretical basis for policy-making to improve adolescent students' seat belt use.

## Introduction

In low and middle-income countries, the rate of road traffic-related deaths is high ^1^. Road Traffic Injuries (RTIs) have a significant effect on the health and economy of societies, individuals, and families ^2^. In 2019, the number of RTIs and deaths in Iran was 16,946 and 347,307 people, respectively ^3^. The human, environmental, and vehicle-related factors are the main causes of RTIs ^4^. Human factors, such as seat belt and helmet wearing behaviors, ignoring traffic regulations and rules, and illegal speeding are the important causes of the RTIs^5,6^. Safety belt wearing is one of the most effective means of reducing fatal and nonfatal injuries in motor vehicle crashes. Previous studies have reported that seat belt use has an important role in reducing the number and severity of injuries^7,8^. The use of seat belts decreases the probability of death or severity of RTIs. A seat belt prevents throwing out from the vehicle, distributes forces of a crash over a wide surface of the body, declines the speed of falling, and protects the head and spinal cord from serious injury ^9^. {Administration, 2001 #10}According to previous studies, the rate of seat belt use was low among men, young adults, obese people, and rear seat occupants ^10-14^{Axelsson, 2019 #17}. {Administration, 2017 #12}Despite the increasing rate of seat belt use in adults, the rate of increase is low in adolescents ^15^. Adolescents break ties with childhood and are fascinated by gaining independence, and also due to the lack of emotional and cognitive maturity, perform risky behaviors, compared to the adults ^16,17^. Therefore, further studies are needed to find reasons for the low rate of seat belt use and solutions to improve it among adolescents. There are limited studies on seat belt-wearing behavior among adolescents, especially as car occupants. This study assessed seat belt use behavior among adolescent students (12-14 years old) as front- or rear-seat car occupants outside or inside the city.

Theory of Planned Behavior (TPB) is a suitable social psychological theory that has been generally applied to address beliefs, values, and attitudes that affect an extensive range of health behaviors ^18^.{Şimşekoğlu, 2009 #40} Moreover, it is an appropriate theory for explaining the seat belt use behavior of drivers and car occupants^19-21^. In a study among university student passengers in the age group of 16-25 years, the capability of TPBin predicting seat belt use behavior among students as front and rearseat passengers has been reported ^22^. Concerning the lack of emotional and cognitive maturity in adolescents, improving the belief and attitude to the suitability of seat belt use in reducing the severity of injuries in motor vehicle crashes could be helpful; therefore, the TPB model was also used in the present study. The results of the present study can demonstrate the importance of the educational program of seat belt use in promoting adolescent attitude and intention on seat belt use as the occupant.

**Figure 1:**
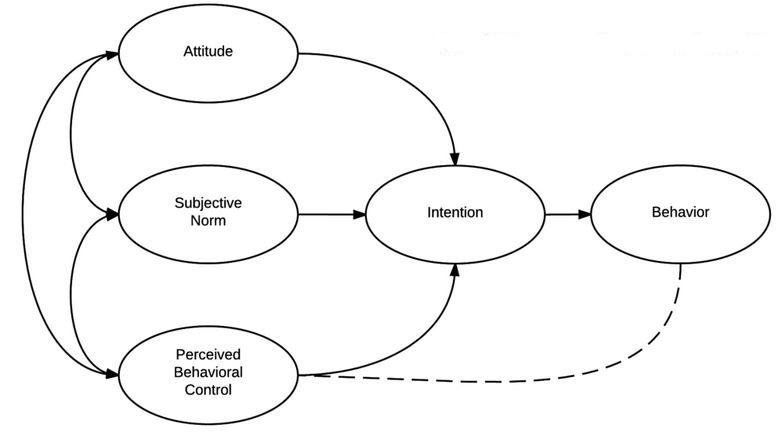
Diagram of the Theory of Planned Behavior

According to the TPB and as indicated in Figure 1, attitude is a favorable or unfavorable appraisal of a given behavior. The subjective norm is a social pressure to behave or not behave in a certain manner. In addition, perceived behavioral control is referred to as people's perception of the ease or difficulty of performing the behavior. Behavioral intention is the motivational factor that affects behavior determined by three factors, namely attitudes, subjective norms, and perceived behavioral control. Moreover, the behavior is determined by intention and perceived behavioral control{Ajzen, #23} ^23^.

As can be inferred, the rate of seat belt wearing among adolescents is lower, compared to that in adults; therefore, more studies are needed to assess the factors controlling this phenomenon. Furthermore, it has been confirmed that the TPB model is suitable for examining seat belt use behavior ^20^. Accordingly, this study aimed to investigate the predictors of seat belt-wearing intention and behavior based on the TPB.

## Methods

###  Study design and participants

 This cross-sectional study was conducted from 30 November 2019 to 5 January 2020 on 952 adolescent school students, and the sample size was determined using the following formula:


$$n=\frac{{\left(z_{\alpha /2}\right)}^{2}p\left(1-p\right)}{E^{2}}$$

Where

α=0.05

and

p=0.51

denote the prevalence of the seat belt use, which was determined based on the previous studies^24^, and

E=0.08p.

**Figure 2:**
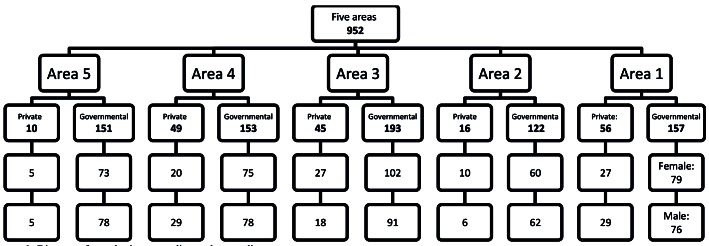
Diagram of sample sizes according to the sampling structure

Moreover, a cluster sampling correction coefficient of 1.5 and a 10% attrition rate were considered; therefore, the final sample size was considered to be 952 (Figure 2). The population of this study included 445 boys and 497 girls, who were selected through the cluster random sampling method. The students were 13-15 years old and studying in academic grades of 7, 8, and 9 in junior high schools of Tabriz, a city with five educational zones located in the northwest of Iran. First, the list of junior high schools was received from the department of education. Accordingly, a total of 20 schools (four schools in each region; two public schools and two private schools) were selected from the mentioned districts or zones. One classroom from each grade (7, 8, and 9) was selected in each school, and then, several students were chosen from each classroom through the simple random sampling method based on the sample size. The students were informed that their participation in the study was voluntary. The oral informed consents were obtained from the students, and the written informed consents were acquired from students' parents.

###  Inclusion criteria

 The students who were studying in junior high schools in the 7, 8, and 9 academic grades and were willing to participate in the study if their parent's consent for their children's participation could be obtained were included in the present study.

###  Data collection instrument

The data were collected using a researcher-made questionnaire that was developed based on previous studies^25^, {Wermert, 2012 #33}^26^. The questionnaire consisted of two parts; the first part included questions about demographic characteristics, such as age, gender, and educational level, and the second part consisted of 76 questions that focused on the constructs of the TPB to measure the determinants of seat belt fastening behavior among students in various dimensions. Regarding the constructs of TPB, the questions were about attitude, subjective norms, perceived behavior control, intention, and seat belt use behaviors of the students. The questions on attitude, subjective norms, perceived behavior control, and intention were rated on a 5-point Likert scale ranging from 1 (strongly agree) to 5 (strongly disagree). The questions on attitude consisted of the behavioral beliefs with seven questions (e.g., "fastening the seat belt prevents me from falling and being injured during a crash") and evaluations of behavioral outcomes with seven questions (e.g., "it is important for me to avoid falling and injuring myself during a crash"). In addition, the questions on subjective norms consisted of the normative beliefs with eight questions (e.g., "my closest friend emphasizes that I should fasten my seat belt every time I get in the car") and motivation to comply with eight questions (e.g., "my closest friend's emphasis on wearing a seat belt is important to me"). The perceived behavior control consisted of control beliefs with nine questions (e.g., "fastening the seat belt makes me feel suffocated") and perceived power with nine questions (e.g., "feeling suffocated causes me not to wear a seat belt when I am riding a car"). Moreover, behavioral intentions included four questions (e.g., "I intend to use a seat belt when riding in the car as a front-seat occupant in the city") and the behaviors consisted of four questions (e.g., "I fasten my seat belt when I sit in the front seat of the car in the city"). These questions were scored based on a 5-point Likert scale for using the seat belt as follows: 5 (always), 4 (most of the times), 3 (sometimes), 2 (seldom), and 1 (never). The total score for the behavior variable was calculated as the average of all the questions related to the behavior, and its value was obtained between 1 and 5.

 The Content Validity Index (CVI) and Content Validity Ratio (CVR) were used to evaluate the data collection tool in terms of content and face validity. The opinions of 12 specialists in health and safety promotion, health education, and traffic were sought. The CVI and CVR for all the dimensions of the questionnaire were higher than 9.0 and 0.8, respectively.

 Furthermore, for determining the reliability of the questionnaire, it was filled out by 50 junior school students in Tabriz, Iran. It should be noted that they were not included in the original sample recruitment. The Cronbach’s alpha coefficients were estimated for the dimensions of the questionnaire including attitude (behavioral beliefs: 0.70; 95% CI: 0.69, 0.73 and evaluations of behavioral outcomes: 0.73; 95% CI: 0.72, 0.76); subjective norms (normative beliefs: 0.82; 95% CI: 0.81, 0.74 and motivation to comply: 0.89; 95% CI: 0.88, 0.901); perceived behavior control (control beliefs: 0.83; 95% CI: 0.82, 0.85 and perceived power: 0.85; 95% CI: 0.84-0.87); behavioral intention: 0.78; 95% CI: 0.77, 0.80; and behaviors: 0.75; 95% CI: 0.74, 0.78.

 The questionnaire was distributed among students after coordination with the education office authorities in the province and schools. The researcher provided the necessary explanations on how to answer the questions, and the self-reporting method was used to gain participants' responses. The participants' names were not recorded in the questionnaire, and other information was kept confidential and used only for this study. It is noteworthy that among 952 students who were randomly selected according to the sample size, 942 students participated in the study, and only 10 participants did not answer the questionnaire (the response rate was approximately 99%) and did not participate in the study due to parental dissatisfaction.

###  Statistical analysis

 Data were analyzed using descriptive and inferential statistics via the SPSS software (version 16). For continuous variables, mean and standard deviation (SD) were used. Moreover, the Pearson correlation coefficient was used to investigate the correlations between variables. In addition, confirmatory factor analysis was applied to validate the structure of the model. Moreover, a linear regression model was built to predict the dimensions of TPB, and a p-value less than 0.05 was considered statistically significant.

####  Ethical Considerations

 The study protocol was approved by the Ethics Committee of Hamadan University of Medical Sciences, Hamadan, Iran (Ref. No: IR. UMSHA. REC.1397.819). The students were informed about the research and their voluntary participation in the study; moreover, oral and written informed consent forms were obtained from the students and their parents. The questionnaires were anonymous, and other data were kept confidential and used only for this study.

## Results

The mean ±SD age of the students was obtained at 13.42 ±1.01 years, and the majority of them were female (53.20%). Regarding the educational level, 36.0%, 34.2%, and 30.8% of the students were studying in grades 7, 8, and 9, respectively. In addition, the results of this study revealed that the mean score of the variable of seat belt use in the front seat inside the city was lower, compared to that outside the city (Table 1). The results of Table 2 indicate that the mean score of perceived behavioral control, subjective norm, attitude, and intention among adolescent students were 56.16 (SD=109.77), 47.10 (SD=119.87), 29.43 (SD=115.97), and 3.96 (SD=0.77), respectively.

**Table 1:** Mean and standard deviation of seat belt-wearing behavior among teenager students

**Covariates**	**Mean**	**Standard Deviation**	**Range**
Fastening the seat belt in the front seat inside the city	3.98	0.75	1-5
Fastening the seat belt in the front seat outside the city	4.47	0.77	1-5
Fastening the seat belt in the rear seat inside the city	3.86	0.69	1-5
Fastening the seat belt in the rear seat outside the city	3.96	0.77	1-5
Behavior	4.06	0.55	1-5

**Table 2:** Mean and standard deviation of perceived behavioral control, subjective norm, attitude, and intention among teenager students

**Covariates**	**Mean**	**SD**	**Range**
Perceived behavioral control	56.16	109.77	9-225
Subjective norm	47.10	119.87	8-200
Attitude	29.43	115.97	7-175
Intention	3.96	0.77	4-20

The correlations among model structures were provided in Table 3. All the correlations were positive and statistically significant (*P*<0.001). Moreover, the results of confirmatory factor analysis were provided in Table 4. Goodness of fit criteria of the model were assessed using several criteria (RMSE=0.043; Chi2/df=2.745; GFI=0.881; PGFI=0.733; AGFI=0.857; CFI=0.939; NFI=0.908; and TLI=0.930). The acceptable cut-points of the above criteria for the goodness of fit of a model are RMSE<0.08, Chi2/df <3, GFI> 0.8, PGFI>0.5, AGFI>0.8, CFI>0.8, NFI>0.8, and TLI>0.8, respectively so that all criteria were in the acceptable range.

**Table 3:** Pearson correlation coefficient between latent variables

**Construct**	**Attitude**	**Subjective** **norm**	**Perceive behavioral** **control**	**Behavior**	**Behavioral** **intention**
Attitude	1.000				
Subjective norm	0.671	1.000			
Perceived behavioral control	0.404	0.372	1.000		
Behavior	0.569	0.628	0.422	1.000	
Behavioral intention	0.587	0.580	0.384	0.571	1.000

**Table 4:** Regression analysis between the main constructs of the Theory of Planned Behavior

**Construct**	**Relationships**	**Construct**	**Estimate**	**SE**	**CR**	***P*** **-value**
Behavioral intention	!	Attitude	-0.838	0.056	-15.089	0.001
Behavioral intention	!	Perceived behavioral control	-0.062	0.026	-2.373	0.018
Behavioral intention	!	Subjective norm	1.000^a^	-	-	-
Behavior	!	Behavioral intention	0.782	0.050	15.766	0.001
Behavior	!	Perceived behavioral control	0.035	0.006	5.897	0.001
Attitude	1	Subjective norm	20.823	1.472	14.143	0.001
Attitude	1	Perceived behavioral control	11.830	1.159	10.209	0.001
Perceived behavioral control	1	Subjective norm	14.518	1.425	10.192	0.001

^a^The parameter for this construct was set to 1 to satisfy identifiability condition.

According to Table 4, one-way relationships were observed among the dimensions of behavioral intention-attitude, behavioral intention-perceived behavior control, behavior-behavioral intention, and behavior-perceived behavioral control (*P =*0.005). Moreover, the results of the covariance test showed that all two-way relationships among the studied structures were significant (*P*=0.005).

A regression analysis was then conducted to investigate the relationship between dependent and independent variables. Table 5 indicates the regression analysis results to specify the determinants of the intention of seat belt-wearing behavior. The constructs of perceived behavioral control (β=0.137; 95% CI: 0.006, 0.013; *P*<0.001), subjective norm (β=0.313; 95% CI: 0.021, 0.032; *P*<0.001), and attitude (β=0.322; 95% CI: 0.034, 0.053; *P*<0.001) had a significant and positive relationship with the mean of the intention of seat belt-wearing behavior. In this respect, with a one-unit increase in attitude, 0.044 units of the intention of seat belt-wearing behavior would increase. Furthermore, with a one-unit increase in the subjective norm, 0.027 units of the intention of seat belt-wearing behavior would increase. Finally, with a one-unit increase in perceived behavioral control, 0.010 units of the intention of seat belt-wearing behavior would increase. For the regression mentioned above, multicollinearity was assessed using "condition index" according to the eigenvalues. Therefore, these values were 5.34, 7.37, and 13.18 for the three included variables, namely attitude, subjective norms, and perceived behavioral control, respectively, which were lower than the cut-off point of 15. Therefore for this model, there was no serious multicollinearity. In addition, the VIF for all the three variables was less than 10 (1.91, 1.86, and 1.22 for the three variables, respectively). Autocorrelation between residuals was assessed using Durbin-Watson, which was d=1.942, indicating no serious autocorrelations between the residuals. Moreover, the Breusch-Pagen test did not show a violation of the homoscedasticity assumption (*P*=0.141). The normality of the residuals was assessed using the Kolmogorov-Smirnov test statistic (*P*=0.643), and the linearity assumption was checked visually using scatter plots.

**Table 5:** Regression analysis to predict the intention of seat belt-wearing behavior based on the constructs of Theory of Planned Behavior

**Model 3**	**Unstandardized Coefficients**		**Standardized Coefficients**	**T**	***P*** **-value**
	**β**	**SE**	**Beta**		
Intercept	6.032	0.406		14.85	0.001
Attitude	0.044	0.005	0.322	9.41	0.001
Subjective norms	0.027	0.003	0.313	9.30	0.001
Perceived behavioral control	0.010	0.002	0.137	5.02	0.001

The results presented in Table 6 indicate the severity and direction of the impact of independent variables on dependent variables. The mean of perceived behavioral control had a significantly positive relationship with the mean of seat belt-wearing behavior (β=0.422.; 95% CI: 0.030, 0.037; *P*<0.001). In this regard, a one-unit increase in the mean of perceived behavioral control leads to a 0.034-unit increase in the mean of seat belt-wearing behavior. According to the obtained results, the impact degree of mean perceived behavioral control on the mean of seat belt-wearing behavior was equal to 0.422. Moreover, autocorrelation between residuals was assessed using Durbin-Watson, which was d=1.813, indicating no serious autocorrelations between the residuals. Moreover, the Breusch-Pagen test did not show a violation of the homoscedasticity assumption (*P*=0.421), and the normality of the residuals was assessed using the Kolmogorov-Smirnov statistic test (*P*=0.372).

**Table 6:** Regression analysis to predict seat belt-wearing behavior based on the perceived behavioral control

**Model 1**	**Unstandardized Coefficients**		**Standardized Coefficients**	**T**	***P*** **-value**
	**β**	**Std. Error**	**Beta**		
Intercept	9.263	0.288		32.18	0.001
Perceived behavioral control	0.034	0.002	0.422	14.36	0.001

The results also revealed that the predictor variable of the intention of seat belt-wearing behavior with the value of 0.571 correlated with seat belt-wearing behavior. The mean of the intention of behavior (β=0.571; 95% CI: 0.62, 0.64; *P*<0.001) had a significantly positive relationship with the mean of seat belt-wearing behavior. Therefore, with a one-unit increase in the intention of behavior, seat belt-wearing behavior would increase by 0.633 units. In addition, the influence of the intention of behavior on seat belt-wearing behavior was equal to 0.571 (Table 7).

**Table 7:** Regression analysis to predictseat belt-wearing behavior based on the behavioral intention

**Model 2**	**Unstandardized Coefficients**		**Standardized Coefficients**	**T**	**P-value**
	**β **	**SE**	**Beta**		
Intercept	3.191	0.470		6.79	0.001
Behavioral intention	0.633	0.030	0.571	21.44	0.001

Autocorrelation between residuals was assessed using Durbin-Watson, which was d=1.903, indicating no serious autocorrelations between the residuals. Moreover, the Breusch-Pagen test did not show a violation of the homoscedasticity assumption (*P*=0.116), and the normality of the residuals was assessed using the Kolmogorov-Smirnov statistic test (*P*=0.223).

## Discussion

The results of the present investigation demonstrated that the seat belt use in the front seat inside the city was lower than that outside the city. The perceived behavioral control, subjective norm, and attitude had a significant and positive relationship with the intention of seat belt-wearing behavior. Moreover, the behavioral intention had a significantly positive relationship with seat belt-wearing behavior. Furthermore, according to the results, it was revealed that the rate of seat belt wearing in front seat occupants was higher than that in rear occupants. In this respect, the results of previous studies were in line with the findings of the present study ^27,28^, and the perception of low risk could be the reason for the low level of seat belt use inside the city.

It can be interpreted from the results that perceived behavioral control, subjective norms, and attitude had a positive relationship with the intention of seat belt-wearing behavior. In line with the result of the present investigation, the previous studies have reported that attitude, subjective norms, and perceived behavioral control significantly predicted the intention of seat belt wearing ^29,30^. In a study among undergraduate front-seat occupant students aged 18-51 years in Brazil, the intention had a significant correlation with all TBP variables, and the highest correlation was found with attitude. Moreover, among rear seat occupants, the intention had a high correlation with attitude and subjective norm ^31^.

In another study among university student passengers with an age range of 16-25 years, the intention had the highest impact on seat belt-wearing behavior in both front and rear seat passengers. In addition, attitude and subjective norms were significant predictors of the intention to use a seat belt ^22^. In contrast to the present study, Simsekoglu and Lajunen (2008) found that perceived behavioral control was not a predictor of either intention or behavior of seat belt wearing. The mean ±SD age of participants was obtained at 21.8±5.0 years^32^.

The subjective norms influence people’s intention and behavior based on social learning theory, observations, and perceptions of how others are generally involved in health behavior ^33^{Svenson, 1985 #41}, ^34^. {Simons-Morton, 2012 #42} Simons- Morton et al. (2012) found that social norms could affect traffic-related behaviors of adolescents^35^. Moreover, Dunlop and Romer (2010) reported that normative perceptions related to seat belt wearing of friends and school peers were correlated with not wearing a seat belt ^36^. The risky behaviors, such as not wearing seat belts, are more frequent among adolescents than adults ^37,39^.{Scott-Parker, 2014 #47}{Li, 2013 #46} The perceived behavioral control could also affect the behavior intention of adolescents. On the one hand, adolescents break ties with childhood and, on the other hand, they are fascinated by gaining independence. Furthermore, due to the lack of emotional and cognitive maturity, they perform more risky behaviors than adults ^16,40^.

According to the results, behavioral intention and perceived behavioral control directly affect seat belt-wearing behavior. In a study, among front-seat occupants, attitude and intention significantly correlated with seat belt-wearing behavior. Among rear seat occupants, perceived behavioral control and intention had a significant correlation with seat belt use behavior ^31^. In this regard, Moeini et al. (2019) showed that the perceived protection motivation could increase the mean score of the skin cancer preventive behaviors ^41^. In addition, Beck et al. (2013) reported that positive beliefs about seat belts could enhance the likelihood of seat belt use ^27^.

Some previous studies indicated that the main reasons for not using seat belts among young adults are forgetfulness/laziness, perceived low risk of injury, and discomfort ^14,42^. In a study on seat belt-wearing behavior among high school adolescents in Qatar, some significant factors affecting this behavior were extracted as follows: (a) being a driver, (b) personal perception of the impact of seat belt wearing in saving lives, (c) keen to require back-seat passengers to fasten seat belt, and (d) previous participation in a traffic safety campaign that emphasized the benefits of seat belt wearing ^43^.

 The improvement of attitudes and subjective norms of seat belt-wearing behavior to increase intention would consequently affect seat belt wearing rates. The intention is a significant variable to explain seat belt wearing. Some educational programs for enhancing positive attitudes on seat belt wearing with the focus on benefits of seat belt use and campaigns to stimulate social disapproval of not using seat belts among adolescents could be suitable in improving the seat belt-wearing behavior. The large sample size of this study can be considered one of its strengths, and applying a self-reported method for data collection can be considered its major limitation.

## Conclusion

 It was revealed that the rate of seat belt wearing among adolescent students as car occupants were low. The TPB is an appropriate theory to determine the predictor factors of seat belt-wearing behavior among adolescent students as car occupants. The perceived behavioral control, subjective norms, and attitude variables had a positive relationship with the intention of seat belt-wearing behavior. Moreover, behavioral intention is directly correlated with seat belt-wearing behavior. The results revealed that the appropriate intervention programs are needed to improve the rate of seat belt wearing intention and behavior among adolescent students.

 Acknowledgments

 The study protocol was approved by Hamadan University of Medical Sciences, Hamadan, Iran, and Tabriz University of Medical Sciences, Tabriz, Iran. The authors would like to thank all the students and their parents who participated in this study.

## Conflict of Interest

 The authors declare that they have no conflict of interests.

## Funding

 This study was supported by Deputy of Research and Technology of Hamadan University of Medical Sciences, Hamadan, Iran [Grant Ref. No. 9711237080] and Tabriz University of Medical Sciences, Tabriz, Iran [Grant Ref. No. 62722]. They had no role in research design, data collection, analysis, and manuscript writing.

 Highlights

- The rate of seat belt use among adolescent students as car occupants in the front seat inside the city was lower than that outside the city.
- The Theory of Planned Behavior is an appropriate theory to determine the predictor factors of seat belt-wearing behavior among adolescents.
- The behavioral intention had a significantly positive relationship with seat belt-wearing behavior among adolescents.
